# Supplementary material for: Behavioral response of insecticide-resistant mosquitoes against spatial repellent: A modified self-propelled particle model simulation
Source: PLoS One. 2020 Dec 29;15(12):e0244447. doi: 10.1371/journal.pone.0244447 (PMC7771694; doi:10.1371/journal.pone.0244447)
Supplement: S1 Fig — Scenarios: A) random walk; B) with attractant alone; C) with repellent and no insecticide resistance; D) with repellent and insecticide resistance; E) with repellent, attractant and resistance assuming β/α = 1.0. Simulation periods: Blue colored section 0–10 s, green colored section 11–30 s and red colored section 31–60 s. (PPTX) [file pone.0244447.s001.pptx]

## Slide 1
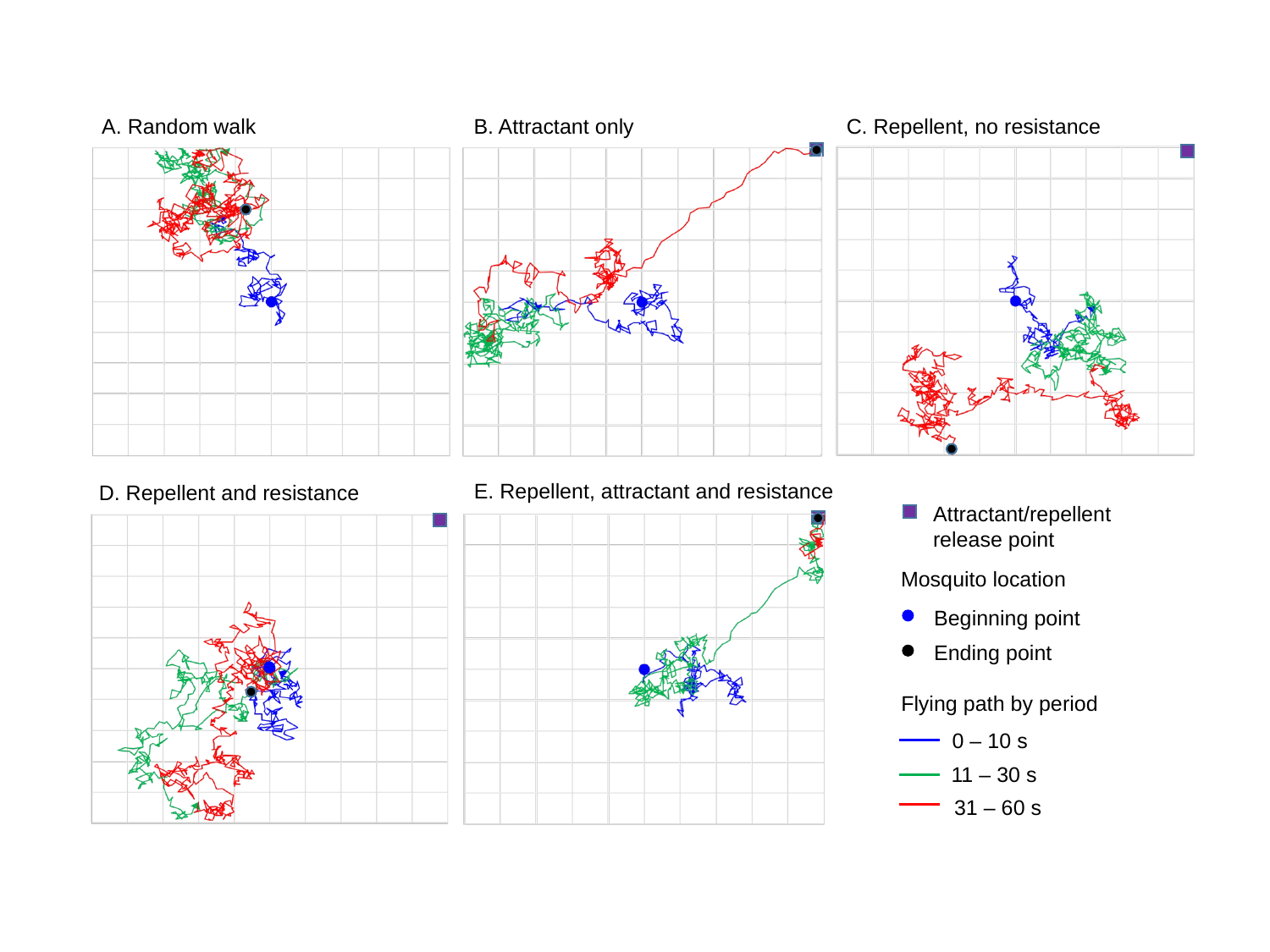

C. Repellent, no resistance
A. Random walk
B. Attractant only
E. Repellent, attractant and resistance
D. Repellent and resistance
Attractant/repellent release point
Mosquito location
Beginning point
Ending point
Flying path by period
0 – 10 s
11 – 30 s
31 – 60 s
